# Bacteriostatic Effect of Piezoelectric Poly-3-Hydroxybutyrate and Polyvinylidene Fluoride Polymer Films under Ultrasound Treatment

**DOI:** 10.3390/polym12010240

**Published:** 2020-01-20

**Authors:** Ivan S. Vatlin, Roman V. Chernozem, Alexander S. Timin, Anna P. Chernova, Evgeny V. Plotnikov, Yulia R. Mukhortova, Maria A. Surmeneva, Roman A. Surmenev

**Affiliations:** 1 Physical Materials Science and Composite Materials Centre, National Research Tomsk Polytechnic University, 634050 Tomsk, Russia; vatlin.i@mail.ru (I.S.V.); bigbbro@yandex.ru (R.V.C.); a_timin@mail.ru (A.S.T.); apa2004@mail.ru (A.P.C.); plotnikov.e@mail.ru (E.V.P.); pheniks-100@rambler.ru (Y.R.M.); surmenevamaria@mail.ru (M.A.S.); 2 First Ivan P. Pavlov State Medical University of St. Petersburg, 197022 Saint-Petersburg, Russia

**Keywords:** piezoelectricity, polymers, piezo-catalysis, bacteriostatic effect, ultrasound

## Abstract

Antibiotic resistance of bacteria stimulates the development of new treatment approaches. Piezoelectric-catalysis has attracted much attention due to the possibility to effectively provide antibacterial effect via generation of reactive oxygen species. However, the influence of the surface charge or potential of a piezopolymer on bacteria has not been sufficiently studied so far. This study reports the fabrication and characterization of thin films of piezoelectric polyhydroxybutyrate, polyvinylidene fluoride, and polyvinylidene fluoride trifluoroethylene as well as non-piezoelectric polycaprolactone polymers fabricated using solution casting approach. The piezoelectric coefficient (*d_33_*) and surface electric peak-to-peak potential generated by the cyclic mechanical stress applied to the films were measured. Neither any toxic effect of the polymer films nor ultrasound influence on *Escherichia coli* bacteria behavior is observed. However, significant inhibition of the growth of bacteria is revealed during mechanical stimulation of piezoelectric samples via ultrasound treatment. Thus, this study demonstrates clear bacteriostatic effect of piezoelectric polymers for different tissue engineering applications.

## 1. Introduction

The appearance of the infections with consequent diseases after surgery is one of the most serious complications [1]. Moreover, the frequent use of antibiotics to treat bacterial infections and diseases promotes the increase of antibiotic resistance of bacteria, which can cause problems to human health [2]. Therefore, the development of new alternative antibacterial agents and methodologies without the use of any antibiotics is required. As a possible approach, the development of composite biomaterials with different nanoscopic antibacterial agents has been widely studied [3,4,5]. Meanwhile, there are alternative ways of using bioactive electrically charged surfaces for the antimicrobial treatment, such as photodynamic therapy (PDT) based on the photoelectric effect takes advantage of photo-induced reactive oxygen species (ROS) generated with photosensitizers [6]. In turn, during the interaction with bacteria, ROS will induce the peroxidation of the polyunsaturated phospholipid component of the lipid membrane and promote the disruption of the cell respiration to destroy bacteria [7]. However, the absence of visible light limits the application of PDT. 

The piezoelectricity is a material property, which is connected with the electric charge formation on the surface in response to applied mechanical stress [8]. The localized electric charge, generated by piezoelectric materials, plays a key role in the healing process of injured or damaged tissues [9]. Hong et al. first demonstrated that the piezoelectric potential can be converted into piezo-chemical potential in aqueous solution catalyzing water decomposition (piezo-catalysis), i.e., generating ROS [10]. Afterwards, numerous studies reported the inactivation of bacteria via piezo-catalysis using piezoelectric ceramics [11,12] as well as inhibiting cancer cell proliferation [13]. However, it is known that ceramics is brittle and lacks required for tissue engineering applications properties compared with polymers [14].

Recently, Ando et al. observed the antibacterial effect of piezoelectricity generated by the deformed polymer yarns [15], which possess significantly lower piezoelectric response compared to ceramics. However, the comparative study of the polymers with different piezoelectric properties has not been reported yet. Therefore, this study is aimed at investigation of the influence of the surface electric potential generated by polymer films on the bacterial growth. Morphology, molecular structure and piezoelectric response of the films based on biodegradable polyhydroxybutyrate, non-biodegradable polyvinylidene fluoride and polyvinylidene fluoride trifluoroethylene polymers as well as their bacterial behavior in dynamic mechanical conditions simulated by ultrasound will be studied. Since these polymers are widely used in diverse biomedical applications, the obtained results are of interest for tissue engineering, e.g., wound healing, bone tissue, etc.

## 2. Experimental Part

### 2.1. Materials and Methods

Polymer powders of polycaprolactone (PCL) (*M*_n_ = 80,000 g × mol^−1^, Sigma Aldrich, St. Louis, MO, USA), or poly[(R)3-hydroxybutyrate] (PHB) (natural origin, Sigma Aldrich, Germany) were dissolved in a chloroform, while poly(vinylidene fluoride) (PVDF) (*M*_n_ = 71,000 g × mol^−1^, Sigma Aldrich, Germany) and poly(*vinylidene fluoride****-****trifluoroethylene*) (Solvene^®^300/P300, Sigma Aldrich, Germany, |*d*_33_| coefficient 23 pC/N at 110Hz according to the manufacturer) were dissolved in a dimethylformamide (DMF, Merck, Darmstadt, Germany). The polymer concentration in the solutions was as follows: PHB–5 wt. %, PCL–5 wt. %, PVDF–20 wt. %, PVDF-TrFE–20 wt. %. The solvent was evaporated at 40 °C. To prepare PVDF films with β-phase exhibiting piezoelectric properties [14], polarization of the films in dry-air sterilizer was carried out at 120 °C and electric field strength of 0.25 MV/m. The films of PVDF-TrFE were used as prepared without any additional poling.

### 2.2. Characterization of the Prepared Samples

The molecular composition of the prepared films was investigated by FTIR spectroscopy using a Nicolet 5700 (Thermo Fisher, Waltham, MA, USA). The surface morphology of the samples was studied using scanning electron microscopy (SEM) using a Zeiss NEON 40 EsB (Jena, Germany). The piezoelectric charge coefficient (*d_33_*) of the polymer films was measured using a Wide-Range *d*_33_ Tester Meter (APC International, Mackeyville, PA, USA). The surface potential was tested using a custom-made set up with an amplifier (gain 5 × 10^4^) and applied mechanical loading at the frequency of 9 Hz [16].

### 2.3. Antibacterial Test

In this study the influence of ultrasound (U/S) and thereof generated piezoelectric charge on the *Escherichia coli* activity was studied using a Branson 1510 (Sigma-Aldrich, St. Louis, MO, USA) with the intensity 9.6 W/cm^2^. The toxicity of the samples on bacteria was investigated under static conditions and U/S treatment. *E coli* strain ATCC25922 was cultivated overnight in 10 mL of the bacterial culture. A volume of 100 µl from the overnight bacterial culture was spread in Luria Bertani. During experiments, the cell density was measured each 10 minutes for 90 min using an OD600 DiluPhotomete spectrophotometer (Thermo Fisher, Waltham, MA, USA). The obtained intensities were used to calculate the relative value of *I*/*I*_0_, where *I*_0_ represented the OD600 obtained at the beginning of the experiments and *I* represented the OD600 at a particular day it was measured. In parallel, we prepared a series of dilutions and sowed the 5th and 6th dilutions on Petri dishes to determine the number of cells. Colony counting was carried out according to the Koch method after 24 hours of incubation at 37 °C. Statistical analysis of the results obtained was performed using one-way analysis of variation (ANOVA) test. The data are presented as the mean ± standard deviation (SD). A value of *p* < 0.05 was considered as statistically significant.

## 3. Results and Discussion

**Figure 1:**
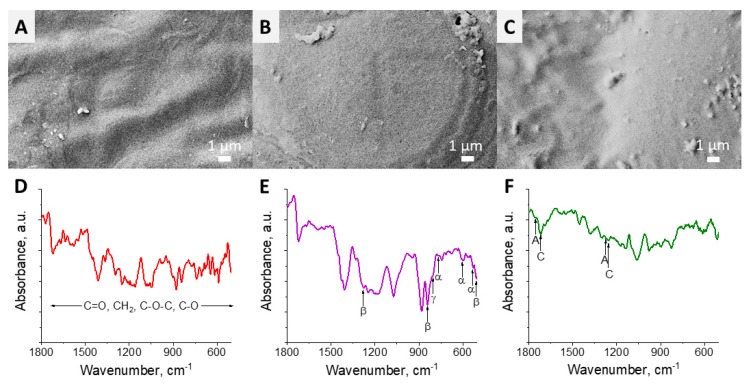
SEM images (upper line) and FTIR spectra (bottom line) of polymer films: (**A**,**D**) polycaprolactone (PCL), (**B**,**E**) poly(vinylidene fluoride) (PVDF) and (**C**,**F**) poly[(R)3-hydroxybutyrate] (PHB).

Figure 1A–C represents SEM images of the fabricated polymer films. A non-porous free from any defects surface was observed for all the samples. Figure 1D–F demonstrates FTIR spectra of the samples in the range 1800–500 cm^−1^. Non-piezoelectric PCL in this range revealed typical peaks such as C=O (at 1727 cm^−1^), C–O–C (at 1233, 1107 and 1042 cm^−1^), C–O (at 1290 and 1160 cm^−1^) stretching and CH_2_ bends (at 1473, 1397 and 1361 cm^−1^) [17]. FTIR spectra revealed crystalline PVDF peaks corresponding to non-electroactive *α*-, both electroactive *γ*- and *β*-phase [18]. Among others, *β*-phase of PVDF and PVDF-TrFE demonstrated the highest piezoresponse [14]. In turn, using the Lambert–Beer law and the peaks at 840 and 763 cm^−1^ assigned to *β*- and α-phase, the fraction of β-phase was estimated as 58% [14]. Piezoelectric PHB films showed all the typical peaks, especially C=O and C–O–C stretching assigned to amorphous (at 1747 and 1270 cm^−1^) and crystalline phases (at 1718 and 1260 cm^−1^) [19].

**Figure 2:**
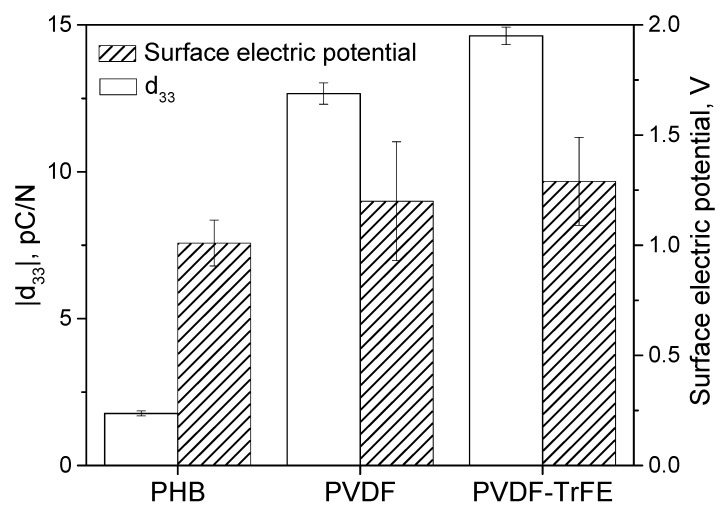
Piezoelectric |*d*_33_| charge coefficient and surface electric potential (peak-to-peak).

The measured |*d*_33_| piezoelectric charge coefficient and surface electric potential for PHB, PVDF-TrFE and PVDF polymer films are shown in Figure 2. The average values of |*d*_33_| and surface electric potential for PHB were similar to that reported in the literature [16]. Both PVDF and PVDF-TrFE films demonstrated higher |*d*_33_| and electric potential values compared to that for PHB. However, the average |*d*_33_| value for PVDF (12.7 ± 0.4 pC/N) was lower than that reported in the study [14]. It can be explained taking into account the reduced *β*-phase content in PVDF in comparison with that reported elsewhere [20]. The increase of the poling temperature and electric field strength can in further improve the crystallinity of PVDF [14]. In the case of PVDF-TrFE, the measured |*d*_33_| coefficient is lower compared to that reported by the manufacturer (23 pC/N at 110 Hz).

**Figure 3:**
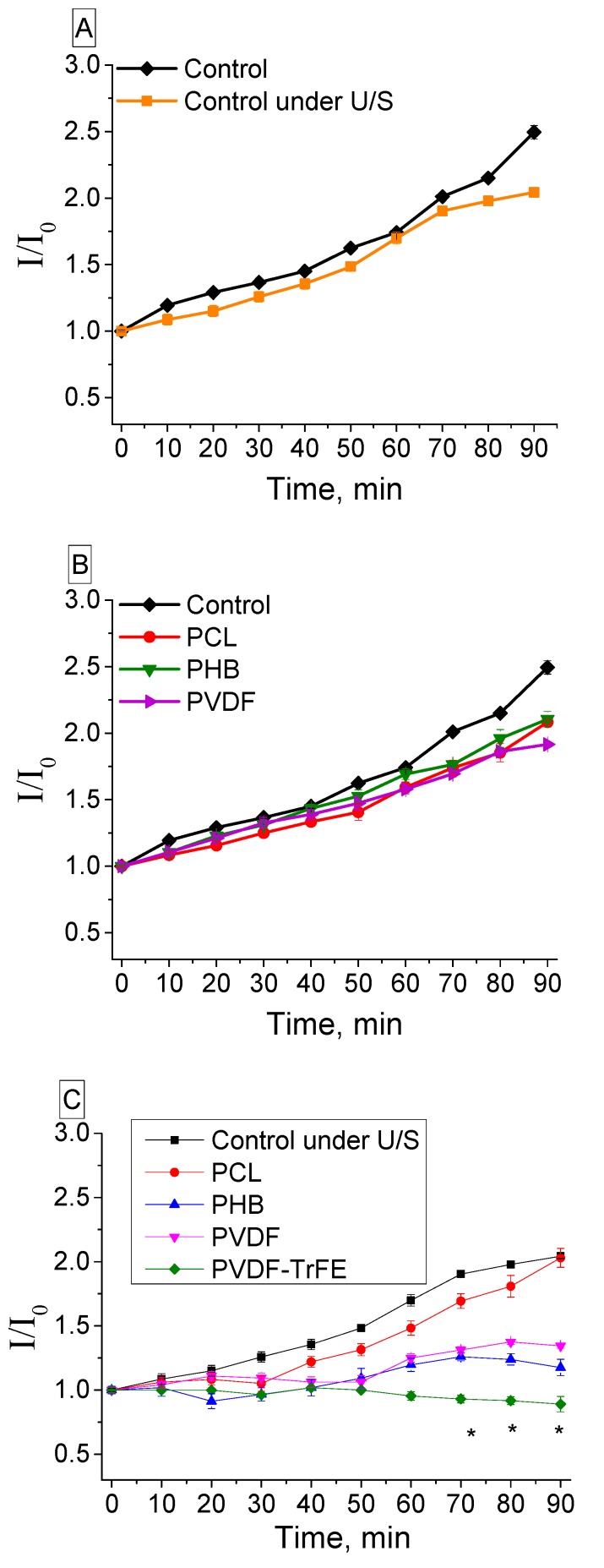
(**A**) Influence of ultrasound on the growth of bacteria: control–bacteria in media, control under U/S–bacteria in media under ultrasound; (**B**) Influence of the samples on bacteria in static conditions; (**C**) *E. coli* growth upon generating piezoelectricity of PVDF, PVDF-TrFE and PHB polymer films. An asterisk (*) denotes a significant statistical difference (*p* < 0.05) estimated by the one-way analysis of variance (ANOVA) between piezoelectric films and non-piezoactive samples (control and PCL films).

The analysis revealed that the bacterial growth rate was slightly slower when U/S was applied to *E. coli* in comparison with the control (Figure 3A). It can be seen that the polymer films are non-toxic for bacteria at the static mechanical conditions (Figure 3B). In turn, a significant statistic effect of the piezoelectricity on *E. coli* was observed (Figure 3C) after 60 min of piezoelectric effect stimulation by U/S, thus resulting in inhibition of the growth of bacteria. However, the decrease of the average optical density (and *I*/*I*_0_ ratio, where *I*_0_ represented the OD600 obtained at the beginning of the experiments and *I* represented the OD600 at a particular day it was measured) of bacteria media already appeared after 40 min of PHB, PVDF and PVDF-TrFE piezoelectric films treated with U/S. As it is mentioned above, the mechanism of the bacteriostatic effect for piezoelectric polymer films is likely connected with the generation of ROS groups [12,13]. However, the piezo-catalysis can even result in bactericidal effect (i.e., killing bacteria), as it was demonstrated with piezo-ceramics [12,13] due to significantly better piezoelectric properties of ceramics compared to polymers [11]. Piezoelectric properties of polymers can be further improved by poling, doping with piezo-inorganic and conductive fillers [14], thus providing enhanced bacteriostatic or even bactericidal effects.

**Figure 4:**
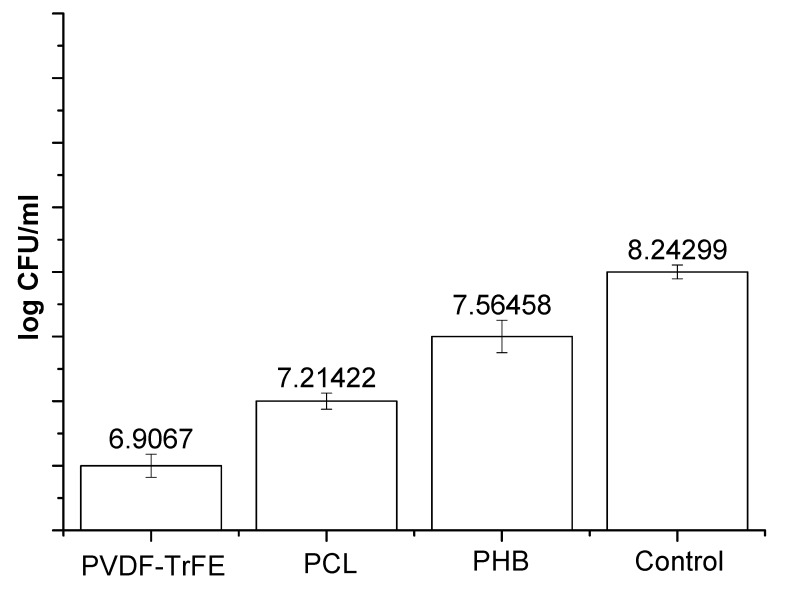
Colony-forming units (CFU) concentration logarithm obtained in the case of ultrasound treatment for 90 min. Control–bacteria in media under ultrasound. Initial concentration of bacteria was 10^9^ CFU. The concentrations of bacteria in the presence of piezomaterials were significantly different from that revealed for control (*p* < 0.05).

The effect of ultrasound treatment on piezoelectric materials immersed in a bacterial suspension leads to a significant decrease in the concentration of bacterial cells (Figure 4). In this study, we found the decrease of the number of bacteria with the presence of the piezoelectric polymer films. The maximum difference was revealed for bacteria in the medium containing a piezoelectric PVDF-TrFE film. The calculated *I*/*I*_0_ ratio (Figure 3) directly correlates with the data obtained using the bacteriological method (Figure 4).

## 4. Conclusions

The polymer films based on PCL, PHB, PVDF and PVDF-TrFE with smooth surface revealed by SEM were successfully fabricated via solution casting approach. No signs of toxic effect of polymer films without U/S treatment were observed. In turn, the inhibition of bacterial growth was shown upon polymer piezoelectricity generated by U/S in case of all studied piezoelectric polymers. Therefore, piezoelectric polymer films demonstrated pronounced bacteriostatic effect, which can be further enhanced and turned to bactericidal effect via fabrication of polymer composites with improved piezoelectric response.
